# Modifying disease registries to address the evolving field in rare diseases: the iSMAc/ITASMAc experience in spinal muscular atrophy

**DOI:** 10.3389/fneur.2026.1833889

**Published:** 2026-06-17

**Authors:** Giorgia Coratti, Chiara Bravetti, Gianpaolo Cicala, Chiara Cutrì, Valeria A. Sansone, Adele D’Amico, Claudio Bruno, Sonia Messina, Federica Ricci, Tiziana Mongini, Michela Coccia, Elena Pegoraro, Riccardo Masson, Angela Berardinelli, Caterina Agosto, Antonella Pini, Antonio Varone, Mara Turri, Massimiliano Filosto, Giacomo Comi, Lorenzo Maggi, Irene Bruno, Maria Grazia D'Angelo, Antonio Trabacca, Veria Vacchiano, Michele Sacchini, Delio Gagliardi, Eustachio D’Errico, Lucia Ruggiero, Lorenzo Verriello, Filippo Brighina, Matteo Garibaldi, Riccardo Zuccarino, Vincenzo Nigro, Roberta Battini, Giulia Ricci, Sabrina Siliquini, Alberto A. Zambon, Betty Polikar, Maria Carmela Pera, Marika Pane, Eugenio Mercuri, ITASMAc Working Group

**Affiliations:** 1 Centro Clinico Nemo, U.O.C. Neuropsichiatria Infantile, Fondazione Policlinico Universitario Agostino Gemelli IRCCS, Rome, Italy; 2 Pediatric Neurology Unit, Catholic University of Sacred Heart, Rome, Italy; 3 The NEMO Centre in Milan, Neurorehabilitation Unit, University of Milan, ASST Niguarda Hospital, Milan, Italy; 4 Unit of Neuromuscular and Neurodegenerative Disorders, Translational Paediatrics and Clinical Genetics, Bambino Gesù Children's Hospital, IRCCS, Rome, Italy; 5 Centre of Translational and Experimental Myology, Department of Neuroscience, Rehabilitation, Ophthalmology Genetics, Maternal and Child Health, IRCCS Istituto Giannina Gaslini, University of Genoa, Genoa, Italy; 6 Department of Clinical and Experimental Medicine, University of Messina, Messina, Italy; 7 Section of Child and Adolescent Neuropsychiatry, Department of Public Health and Paediatric Sciences, University of Turin, Turin, Italy; 8 Neuromuscular Unit, Department of Neurosciences 'Rita Levi Montalcini', University of Turin, Turin, Italy; 9 NeMO Clinical Centre Ancona - Azienda Ospedaliero-Universitaria Delle Marche, Ancona, Italy; 10 Dipartimento di Salute della Donna e del Bambino, Azienda Ospedale Università Padova, Italy; 11 Department of Pediatric Neuroscience, Fondazione IRCCS Istituto Neurologico 'Carlo Besta', Milan, Italy; 12 C. Mondino Foundation, Pavia, Italy; 13 Dipartimento Di Salute Della Donna E del Bambino, Università Di Padova, Padua, Italy; 14 UOC Clinica Neurologica, IRCCS Istituto Delle Scienze Neurologiche Di Bologna, Bologna, Italy; 15 Pediatric Neurology Unit, Department of Neurosciences, Santobono-Pausilipon Children's Hospital, Naples, Italy; 16 Dipartimento di Neurologia, Stroke Unit, Ospedale di Bolzano, Bolzano, Italy; 17 Department of Clinical and Experimental Sciences, University of Brescia and the NeMO-Brescia Clinical Centre for Neuromuscular Diseases; ERN Euro-NMD Centre ASST Spedali Civili, Brescia, Italy; 18 Neurology Unit, Fondazione IRCCS ca' Granda Ospedale Maggiore Policlinico, Milan, Italy; 19 Neuroimmunology and Neuromuscular Disease Unit, Fondazione IRCCS Istituto Neurologico Carlo Besta, Milan, Italy; 20 Institute for Maternal and Child Health, IRCCS, Burlo Garofolo, Trieste, Italy; 21 Unit of Rehabilitation of Rare Diseases of the Central and Peripheral Nervous System, Scientific Institute IRCCS Eugenio Medea, Bosisio Parini, Italy; 22 Scientific Institute IRCCS “E. Medea”, Scientific Direction, Bosisio Parini, Italy; 23 Centro Clinico NeMO, IRCCS Istituto Delle Scienze Neurologiche Di Bologna, Bologna, Italy; 24 Metabolic Diseases and Neuromuscular Disorder, AOU Meyer-IRCCS, Firenze, Italy; 25 Pediatric Neurology Unit, Pediatric Hospital "Giovanni XXIII", Bari, Italy; 26 Department of Neurology "L. Amaducci", AOU Consorziale Policlinico, Bari, Italy; 27 Department of Neurosciences, Reproductive and Odontostomatological Sciences, University of Naples Federico II, Naples, Italy; 28 Neurology Unit, Department of Head Neck and Neurosciences, Santa Maria della Misericordia University Hospital, Udine, Italy; 29 Department of Biomedicine, Neuroscience and Advanced Diagnostics (BiND), University of Palermo, Palermo, Italy; 30 Neuromuscular and Rare Disease Centre, Department of Neuroscience, Mental Health and Sensory Organs (NESMOS), SAPIENZA University of Rome, Sant'Andrea Hospital, Rome, Italy; 31 NeuroMuscular Omnicentre (NeMO) Trento, Azienda Sanitaria Universitaria Integrata del Trentino (ASUIT), Trento, Italy; 32 Department of Precision Medicine, University of Campania "Luigi Vanvitelli", Naples, Italy; 33 Department of Developmental Neuroscience, IRCCS Fondazione Stella Maris, Pisa, Italy; 34 Department of Clinical and Experimental Medicine, University of Pisa, Pisa, Italy; 35 Child Neuropsychiatry Unit, Paediatric Hospital G Salesi, Ancona, Italy; 36 Neurology Unit, IRCCS San Raffaele Scientific Institute, Milan, Italy; 37 Child Neuropsychiatry Unit, Department of Medicine and Surgery, University of Parma, Parma, Italy

**Keywords:** disease registry, longitudinal data, neuromuscular disorders, real-world data, spinal muscular atrophy

## Abstract

**Introduction:**

The availability of disease modifying therapies in Spinal Muscular Atrophy (SMA) has highlighted the need for more structured registries for collecting reliable information both in treated and untreated individuals. Regulators recommendations have recently become available suggesting how registries should be designed or adapted for different purposes. These include the study of disease epidemiology/natural history in untreated individuals or the assessment in a real world setting of post treatment long term efficacy and safety in much wider cohorts than those included in pivotal trials. We report the adaptation of an academic registry to a nationwide registry describing the different steps of the process.

**Methods:**

All Italian centres were asked to participate. The initial electronic case report form was adapted to be more compliant with recent regulators recommendations, including a more structured coding of adverse events. Training sessions were performed to guarantee data reliability. The governance system was also reviewed and integrated to optimize transparency, accuracy, and collaboration among stakeholders.

**Results:**

All centres agreed to be part of this effort and completed training sessions. A critical analysis of the performance of the registry shows a high compliance of the centres.

**Discussion:**

Our experience suggests that it is possible to keep the reliable structure of an academic registry even when enlarging the registry at national level and adopting a more structured approach in compliance with regulators recommendations. Such a registry can be a valuable source for research purposes and to inform decisions both in a clinical setting and at regulatory level.

## Introduction

In the last two decades the approach to spinal muscular atrophy (SMA) has shifted from largely descriptive practice to an increasingly interventional discipline, especially after the arrival of disease-modifying treatments that have altered disease course (1). The near-simultaneous advent of three disease modifying treatments (DMTs) including gene transfer and other SMN-upregulating molecules (2–6), combined with newborn screening (7, 8), has largely reshaped clinical trajectories and made longitudinal monitoring indispensable. As a result, there has been an effort to create new registries to overcome many of the challenges of the old data infrastructures—centre-specific spreadsheets, non-harmonized databases, and registries with heterogeneous variable definitions and measure possible changes with or without treatment, comparably across centres and over time (9).

The new registries have been aiming to capture such changes, using outcome measures routinely used both in research practice and clinical trials (10–14). Our group has contributed to the development of the International SMA collaboration registry (ISMAR), a disease registry originally developed as part of an international collaboration (ISMAC) across United States, United Kingdom and Italy including a selected number of academic centres in each country (13). The new registry had several advantages. Data quality was more actively designed, focusing on a shared language and on improving standardization by agreeing on outcome definitions, scoring instructions, equipment calibration, and structured data-entry schemas with controlled versioning. Particular attention was paid to regular training of the examiners with inter-rater exercises, refresh modules, and documented competencies for clinicians, physiotherapists, and data managers. ISMAR has been successfully used over the last 7 years collecting information on both treated and untreated SMA individuals and contributing to the literature both on natural history and real-world data in treated individuals (15–24).

There have recently been attempts to further define the scope of these registries. The primary objective of ISMAR was an academic effort limited to highly specialized academic centres that had been involved in clinical trials and early access programs and were able to collect high quality data. This model has proved to be very effective, as all centres had capabilities and resources to collect a large amount of information on each individual SMA patient. The evolving scenario of drug availability has however highlighted the need to adapt the registry.

Since the DMTs were approved and could be prescribed by a much wider number of centres across the countries, there has been a sensible decentralization of care with many individuals opting to be treated and receive routine follow-up closer to home (25, 26).

When we conducted a survey at national level including all the centres identified by the government as prescribing centres for SMA (26), we detected that while although over 50% of the global number of SMA individuals in Italy were followed in the 5 academic centres in ISMAR, the remaining individuals were followed in a much wider number of centres (n = 26) with different levels of previous expertise in SMA and availability of resources. This has led to a revision of the platform and to an expansion of the Registry from its initial configuration to a comprehensive national platform. This was driven by the convergence of several critical factors in the evolving landscape of SMA research and clinical care. Substantial barriers to aggregation of patient data at the national level are related to possible heterogeneity of existing data collection systems across various neuromuscular centres with different research and clinical settings. Recognition of this limitation led to the strategic decision to expand the unified platform architecture used for iSMAc so that could accommodate the diverse clinical workflows, institutional requirements, and technical capabilities present across participating centres throughout Italy, while maintaining standardized data definitions and collection procedures that would ensure comparability of information regardless of the originating site.

The need to develop such a platform was also driven by parallel fundamental shifts in regulatory expectations regarding the role of real-world evidence in supporting therapeutic decision-making and regulatory evaluation. Both regulatory authorities and pharmaceutical sponsors demonstrated increasing recognition that randomized controlled trials, while remaining the gold standard for establishing efficacy and safety, provide limited insight into treatment outcomes in the broader patient population encountered in routine clinical practice. The European Medicines Agency (EMA) has formalized this perspective through a series of guidance documents, including the Guideline on Registry-Based Studies, the Data Quality Framework for Real-World Data (RWD), and guidance on the use and qualification of real-world evidence (RWE) for regulatory decision-making. These documents outline the requirements for data quality, governance, and methodological robustness necessary for registries to be considered fit for regulatory purposes (27–29). For SMA in particular, where multiple disease-modifying therapies have achieved regulatory approval within a compressed timeframe and where treatment decisions increasingly involve complex considerations of timing, sequencing, and combination strategies, the need for robust real-world evidence to contextualize trial findings within large, representative cohorts has become particularly acute (30).

The new version of the Italian registry, ITALIAN SMA Registry (ITASMAR) was therefore designed not merely as an epidemiological tool for natural history characterization, but as a platform capable of generating the high-quality longitudinal data increasingly required by regulatory authorities and sponsors to inform therapeutic development, market access decisions, and clinical guideline formulation in this rapidly evolving therapeutic landscape. Here we report the adaptation of the original ISMAR and the transition to ITASMAR to include all the additional SMA centres in Italy, describing the criteria used for the adaptation and new data from the registries including all patients at national level.

## Materials and methods

This paper reports the process to adapt the original ISMAR to be suitable for data collection at national level. After obtaining extra funding from the companies that were already supporting ISMAR, the process included a number of steps, from the identification of all the centres in Italy to the adaptation of the eCRF, to a critical analysis of the data collected. This was part of larger ongoing study assessing long term changes in disease course in SMA approved by the Ethics Committee of Fondazione Policlinico Universitario A. Gemelli IRCCS (21/02/2018N. 0007592/18).

### Identification of the SMA centres in Italy

The Italian health system is organised into regional governments and each of them identifies one or more as specialist centres within the region which are selected to prescribe treatments such as the DMTs used in SMA. The first step of the process was to obtain the list of the centres in all regions. Among these centres, the only criteria used to select centres to be included in the nationwide registry was that they had to follow at least 2 SMA individuals (children or adults).

This approach ensured nationwide coverage, including centres with varying levels of expertise and patient volumes, thereby supporting the representativeness of the registry.

### Involvement/engagement of the centres

All centres were contacted, informed and asked to be part of the project.

### Ethics committee approval

The protocol was amended to include all the new participating sessions, and the new centres submitted the protocol to their local Ethic Committee.

### Governance

To improve transparency, accuracy, and collaboration among stakeholders the Governance System was reviewed and integrated with new activity profiles.

### Amendment of the eCRF

The eCRF was designed as part of an international effort including a very large number of items that had been thought to be relevant to capture a wide range of aspects, paying particular attention to shared definitions, dictionary and format of data elements to ensure standardization, clarity, and consistency of data collection across all participating centres, with a Manual of Operations providing detailed guidance on the procedures for data entry, collection, and management. The eCRF has proved to be very successful in the original international ISMAR project, it includes a very large number of fields but, when reviewed for a more general use, it was felt that not all the centres may have the adequate resources to complete the totality of the fields included in the forms conceived in the original project. While it was agreed that the eCRF should not be changed for consistency with the original project and for the international collaboration, a number of meetings were performed to establish which items should be kept as mandatory and which one could be considered as optional. This distinction between mandatory and optional fields was designed to ensure feasibility across all centres while preserving the ability to collect more granular data where resources allowed.

Further discussion aimed at identifying possible additional items to be more compliant with to the newly available regulators recommendations.

### Dissemination of training

While waiting for Ethic Committee approval all centres received training sessions for both data entry and clinical examiners and clinicians were asked to be compliant with the proposed data dictionary.

### Patients’ enrolment

After receiving Ethic Committee approval all the centres proposed the consent to all the SMA individuals in their centres and, once consent was obtained, enrolled the patients in the registry, completing the eCRF at each visit.

### Monitoring and data quality

ITASMAR implements a multi-layered monitoring and data quality assurance system designed to ensure data integrity, regulatory compliance, and traceability throughout the data lifecycle. This comprehensive approach combines automated technical controls, systematic internal monitoring, and external verification processes to maintain the highest standards of data quality and patient protection.

In line with the EMA Data Quality Framework for real-world data (29), data quality within ITASMAR is assessed across multiple dimensions beyond completeness, including accuracy, consistency, timeliness, and representativeness.

Accuracy is ensured through structured source data verification procedures performed during external monitoring visits, alongside a comprehensive audit trail capturing all data modifications.

Consistency is maintained through automated validation rules embedded in the eCRF and systematic query management processes addressing discrepancies and logical inconsistencies.

Timeliness is monitored through regular tracking of data entry and query resolution intervals across participating centres.

Representativeness is supported by the nationwide inclusion strategy, involving all prescribing centres and minimizing selection bias through broad eligibility criteria and high participation rates.

These procedures were designed to align with current EMA recommendations for RWD quality and registry-based studies.

### Prevention of duplicate entries

Given the multicentre nature of the registry and the possibility that patients may receive care at multiple participating centres over time, preventing duplicate patient entries represents a critical data quality concern. ITASMAR addresses this challenge through implementation of a unique patient identification system designed to recognize when the same individual is being registered at different sites or at different time points. Personal identifying information, including full name, day of birth, year of birth, sex, and place of birth, is captured through a patient demographic form maintained outside the primary registry platform. These demographic elements are processed through an external algorithm that generates a unique internal identifier number specific to each individual patient. When a participating centre attempts to register a new patient in the system, the registry automatically compares the generated unique identifier against all existing patient records. If the system detects that a patient with an identical unique identifier has already been registered, it prevents completion of the duplicate entry and generates an automated system alert. Upon receipt of a duplicate entry alert, the participating centre that attempted the registration contacts the coordinating centre to initiate a data reconciliation process. The coordinating centre reviews the circumstances surrounding the duplicate detection and determines the appropriate resolution. In many cases, duplicate alerts reflect legitimate clinical scenarios such as transfer of patient care from one participating centre to another, continuation of care for a patient who has relocated geographically, or referral patterns in which patients receive specialized services at multiple centres within the network. The coordinating centre works with both the original registering centre and the centre that triggered the duplicate alert to ensure that the patient’s complete clinical history is appropriately maintained within a single registry record while accurately reflecting care provided at multiple sites.

### Audit trail

At the foundation of the registry’s quality assurance system is a comprehensive audit trail mechanism that ensures complete traceability of all data operations while maintaining compliance with the General Data Protection Regulation (GDPR) and other applicable data protection requirements. Every modification to the patient visit records is automatically captured and preserved, including the precise date and time of each change, the identity of the user performing the modification, and the specific content that was altered. The system maintains previous versions of all records in an immutable format, creating a complete revision chain that preserves the entire history of data evolution throughout the study period. This immutability is achieved through technical controls that prevent any user or system administrator from modifying or deleting logged events, thereby ensuring the reliability and authenticity of the audit record.

The audit trail serves multiple critical functions in maintaining data quality. First, it enables retrospective review of data entry and modification patterns, allowing identification of systematic errors or inconsistencies in data collection practices. Second, it provides accountability by linking all data changes to specific users, encouraging careful and accurate data entry. Third, it supports regulatory compliance by demonstrating adherence to Good Clinical Practice (GCP) standards and data protection requirements. Authorized personnel can access audit information through a graphical interface that permits examination of the complete history for individual patients and visits, facilitating targeted investigation when data quality concerns arise.

Access to the registry is controlled through multi-factor authentication requiring personal credentials supplemented by temporary tokens delivered electronically, with password complexity requirements and mandatory periodic renewal. Role-based access control ensures that each user can access only the data necessary for their specific function within the study, implementing the principle of least privilege and reducing the risk of inadvertent data modification or unauthorized access. While these security measures serve primarily to protect patient privacy and data confidentiality, they also contribute to data quality by ensuring that only trained and authorized personnel can enter or modify study data.

### Continuous internal monitoring

The registry employs systematic internal monitoring procedures designed to identify and resolve data quality issues in near real-time. Weekly remote monitoring reports are generated automatically for all participating centres, providing study coordinators with regular oversight of data collection activities across the network. These reports enable prompt identification of patterns such as incomplete data entry, delayed visit recording, or inconsistent data. When missing or inconsistent data entries are detected, are transmitted to appropriate site personnel for resolution, creating a continuous feedback loop that maintains data completeness and internal consistency.

### External monitoring and source data verification

Independent external monitoring provides an additional layer of quality assurance through periodic on-site visits conducted by trained Clinical Research Associates (CRA). Each participating centre receives at least one monitoring visit during which the CRA performs comprehensive verification activities designed to assess the accuracy, completeness, and regulatory compliance of study conduct and data collection. These visits serve the dual purpose of verifying data quality and providing education and feedback to site personnel to improve ongoing data collection practices.

During monitoring visits, the CRA verifies the completeness and organization of essential regulatory documentation at each site, including the Investigator Site File with all required protocol documents, ethics committee approvals, informed consent forms, and documentation of personnel training and qualifications. Review of the delegation log confirms that study tasks are being performed by appropriately trained and authorized personnel. Verification that all enrolled patients have properly executed informed consent and privacy authorization forms ensures that ethical and regulatory requirements for patient participation are being met.

The core of the external monitoring process is source data verification, in which the CRA compares data recorded in the electronic case report form against original source documents maintained at the clinical site. A random sample representing 10% of enrolled subjects at each centre undergoes detailed verification of all details of the visit. Particular attention is directed to the documentation and reporting of serious adverse events, ensuring that all such events are identified, properly documented in source records, and completely and accurately reported in the registry in accordance with regulatory requirements and study protocols.

Following each monitoring visit, the CRA provides the site with written documentation summarizing activities completed during the visit, sites are requested to notify the CRA when corrective actions have been completed and required documentation is available for review, at which point remote verification occurs through telephone or videoconference. Outstanding issues identified during monitoring visits are tracked systematically by the CRA through ongoing follow-up until satisfactory resolution is achieved.

### Critical analysis of the results

A critical analysis of the registry performance was performed to ascertain centre compliance. Data completeness for mandatory fields was systematically assessed by visit type and section, with descriptive statistics calculated including number of variables per section (n), average percentages for complete, missing, and unknown data, and distribution metrics (median, minimum, and maximum values) to characterize the range of data quality across the registry.

## Results

Supplementary Tables 1 and 2 reports details of the eCRF’s items that were kept as mandatory and the ones that were considered as optional.

### Identification of the SMA centres in Italy

After a review with the regional heath governments 37 centres were identified. Eighteen centres were mainly caring for adults and 19 were either exclusively paediatric centres or were caring for both children and adults.

**Figure 1:**
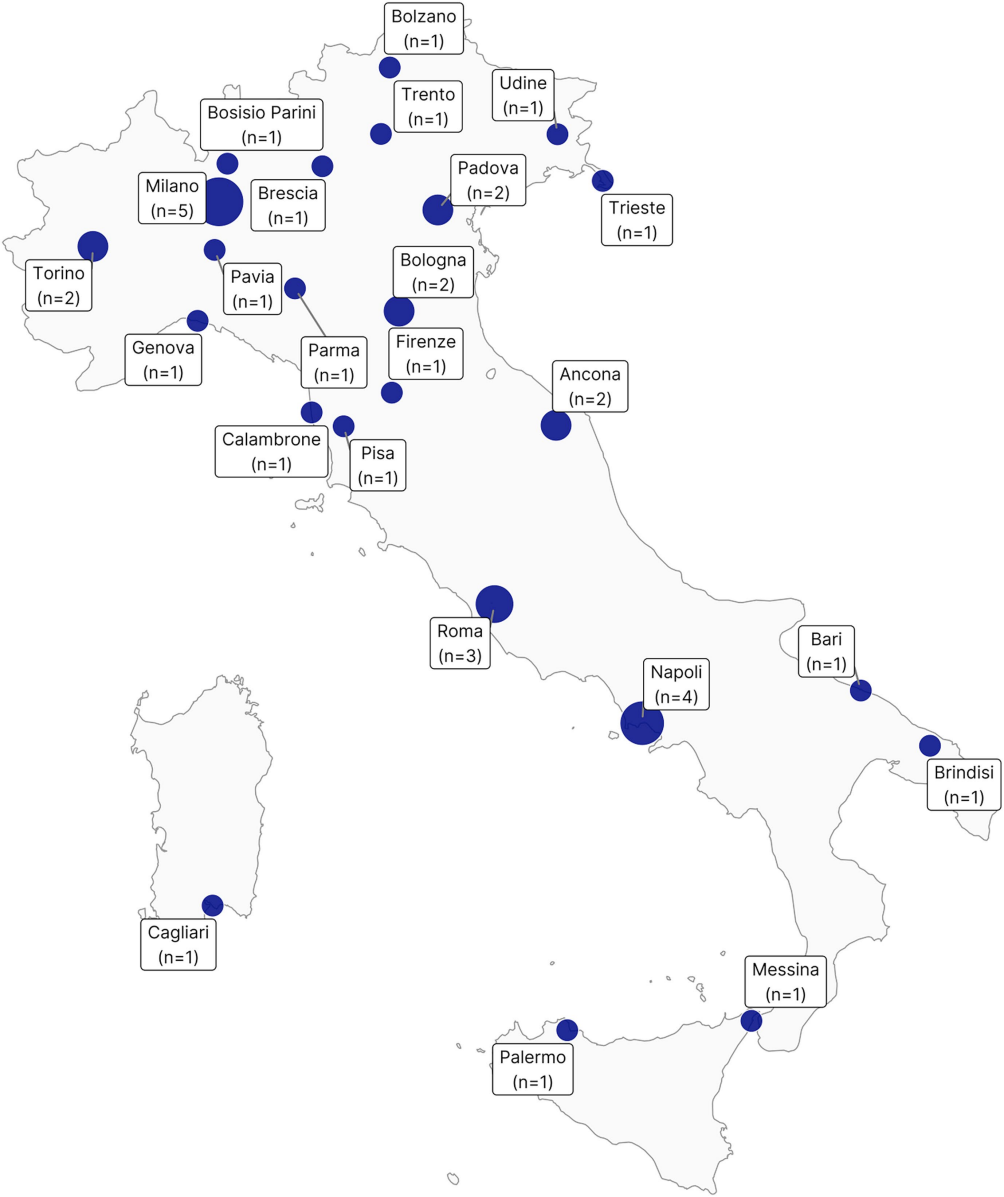
Distribution of the centres.

The distribution of the centres covered all Italian regions. Currently there are 21 centres in the North, 8 in the Centre and 8 in the South of Italy. Figure 1 shows details of the distribution of the centres.

### Involvement /engagement of the centres

All centres contacted and informed of the project agreed to be part of the registry. The great majority of these centres have been part of a previous network assessing natural history since 2006 with examiners fully trained and certified for the functional outcome measures included in the registry. The newly funded collaborative frame including all the centres in Italy was named ITASMAc (ITAlian SMA consortium).

### Ethics committee approval

The main protocol was amended to include all the new participating centres, and the new centres submitted the protocol to their local Ethic Committee.

### Governance

The overall structure of the Governance includes the Executive Committee and the Scientific advisory board. The chair of the steering committee is also the project coordinator. The Executive Committee is the main decision-making and arbitration body and in charge of overall development and oversight of the registry. This includes the Chair, other clinicians with expertise relevant to the project, representatives from patients’ advocacy group and other figures (Ethicists, Legal, Statistician) that are equally crucial for the process of setting guidelines for data ownership, access, and quality assurance and to manage data integrity and address potential data limitations.

The governance also includes dedicated figures for data management, quality assurance and risk management with an external auditor/quality assurance auditor and a Data Protection Officer (DPO) to overlook data protection and privacy. A Chief Operational Officer provides support and guidance on all operational aspects.

The Executive committee regularly receives advice from an external scientific committee for peer reviewing the quality of the research design and the quality of research deliverables. Other external experts may also be invited as *ad hoc* advisors.

### Amendments to the eCRF

There were 2 main additional modifications of the eCRF. The first, based on regulators’ recommendations, was related to the introduction of the medical dictionary for regulatory activities (MedDRA) coding system (31), that allows to have a more structured collection of data related to adverse events, surgeries or procedures, and of coding for medicinal products according to the Article 57 database (32). The other relevant amendment to the eCRF, that is currently being implemented, implies the introduction of additional items for better defining the newborns identified via neonatal screening. In Italy until recently neonatal screening was restricted to a selected number of regions and has progressively been expanded to other regions with the forecast to become available nationwide in the coming year. The additional items also include new functional scales that have been developed in the last few years (33) and provides the possibility to include details of additional neurophysiological assessments or biomarkers, when performed.

**Figure 2:**
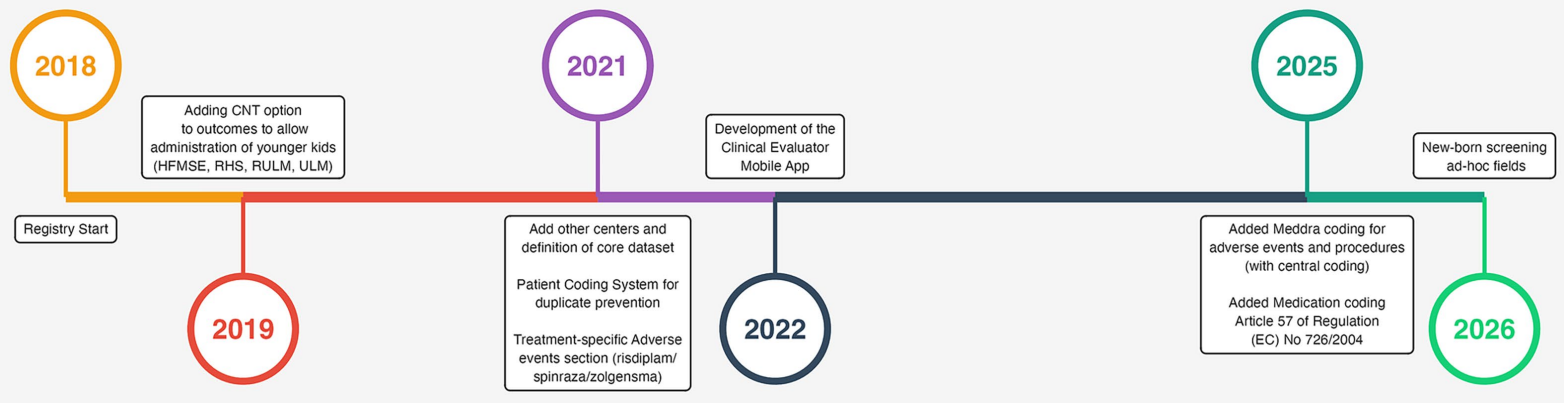
Details of the major changes to the eCRF over the years.

Figure 2 shows details of the major changes to the eCRF over the years.

### Dissemination of training

All centres received training sessions to make them familiar with the agreed eCRF outcome definitions, data dictionary scoring instructions, and the structured data-entry schemas used by the centres already participating in ISMAR.

Centres were also trained on informed consent procedure, storage of the source records and of the documents of the study.

Similarly, clinical observers underwent training with refresh modules of the measures used in clinical routine to ensure inter-rater reliability across examiners.

### Patients’ enrolment

After receiving Ethics Committee approval all the centres proposed the approved version of the consent to the SMA individuals in their centres. Considering that the registry was designed as a disease registry to record all SMA patients in Italy with a genetically confirmed diagnosis of 5q SMA all individuals living with SMA attending the clinics are offered to be in the registry, with no exceptions, irrespective of the SMA type (0-4/presymptomatic), the age, or whether they are treated or not with DMTs or receive other interventions.

At the time of data extraction (November 2025), the ITASMAR registry included 37 participating centres distributed across all Italian regions, comprising 21 centres in Northern Italy, 8 in Central Italy, and 8 in Southern Italy. A total of 1,330 patients with genetically confirmed 5q spinal muscular atrophy were enrolled, representing one of the largest national SMA cohorts in Europe.

The registry includes individuals across the full clinical spectrum (types 0–4 and presymptomatic), irrespective of age or treatment status, with both treated and untreated patients captured in routine clinical practice. This broad inclusion strategy supports the representativeness of the registry with respect to the national SMA population managed in routine clinical practice. Less than 1% of eligible patients declined participation, supporting the broad inclusion and high acceptability of the registry.

Of the 1,330 enrolled patients in the Italian registry, 90 were/are enrolled in clinical trial with experimental drugs, 29 deceased, 44 moved to another country, 20 stopped the follow-up in a tertiary care centre.

### Monitoring and data quality

Real-time validation is performed during the data entry process. Data verification occurs through weekly remote monitoring reports for all centres and automated query generation for missing or inconsistent entries is done weekly. Participating centres demonstrate a strong level of compliance in data entry and a good compliance to timely query resolution (the turn-around time spans from 48 h to 72d, depending on the complexity and the number of queries received). All queries are collected via a Query Log, addressing efficiently and promptly deviations, and preventing the accumulation of unresolved queries (Table 1).

**Table 1:** Query management structure.

Query management specification	Details
Tool Used for Query Management	Query Log – a centralized tool for tracking, categorizing, and resolving data inconsistencies across the network.
Most Common Types of Queries	Data entry errors: typos, missing values, or inconsistencies between fields.
	Date inconsistencies: discrepancies in dates, such as mismatches between admission and discharge or follow-up visit schedules.
	Calculation errors: incorrect calculations or derived values
	Missing mandatory sections/fields required by the CRF or inconsistencies with the protocol’s defined procedures.
Additional Query Reporting Measures	A monthly report system directed to Principal Investigators highlights unresolved queries, compliance levels, and overall query trends, fostering enhanced network transparency.
System Enhancements	Weekly updates to the Query Log ensure efficient handling of increasing data volumes, including automated alerts for overdue or high-priority queries.

### Critical analysis of the results

Data completeness analysis for mandatory fields revealed generally high completion rates across most registry sections (Supplementary Table 1). The majority of sections demonstrated average completeness rates exceeding 90%. The proportion of missing data across all mandatory fields remained consistently low (median <2%), with unknown values varying by section and clinical applicability.

Non-mandatory fields exhibited considerably more variable completeness rates compared to mandatory data elements (Supplementary Table 2), reflecting the optional nature of these assessments. Completeness rates ranged from 34.2 to 100% depending on the section and visit type.

The average proportion of unknown values for non-mandatory fields remained low (<10% for most sections), with missing data representing the primary source of incompleteness.

Consistent with the multidimensional data quality framework described above, accuracy was supported by external monitoring activities including source data verification on a random sample of approximately 10% of enrolled subjects per centre, confirming concordance between source documents and registry data.

Consistency of data was ensured through continuous automated query generation and resolution, with centres demonstrating high compliance in addressing inconsistencies within predefined timelines.

Timeliness analysis showed that query resolution times ranged from 48 h to 72 days, depending on complexity, with most queries addressed within a short timeframe.

## Discussion

The relationship between clinical registries and the wider ecosystem has progressively matured. In Europe, this is reflected in the EMA Guideline on Registry-Based Studies and the EMA Real-World Evidence framework, which emphasize the importance of structured data collection, traceability, auditability, and data quality dimensions for regulatory-grade evidence generation (27, 34–36). This paper illustrates how the infrastructures of a registry can adapt following the advent of therapeutic changes and regulatory recommendations. Until recently registries or infrastructures of data collection have followed mainly two directions. The first disease registries were providing important information on epidemiology or functional changes but were often patient driven with non-harmonized databases collecting information from different centres with heterogeneous variable definitions and centre-specific spreadsheets (37). On the other hand, academic registries, while harmonizing datasets and definition, are often seen by regulators to reflect the activities in specialized tertiary care centres and not necessarily capture ‘real world evidence’ from less experienced centres.

In the last few years, with disease modifying therapies becoming commercially available, the decentralization of care has highlighted the need to combine these two efforts widening the number of centres involved in the registry beyond highly specialized centres while at the same time maintaining the structured format of their registries. With increasing attention from regulators to provide recommendations on the criteria to be used to collect real world data, the challenge faced by clinician and researchers is to maintain high quality data and be compliant with the regulators’ recommendations while expanding data collection beyond highly specialized centres.

In Italy, ISMAC established an academically led disease registry with explicit variable definitions and curation processes (13). The original registry, developed as part of an international academic effort, had a large dataset developed to be fully comprehensive to capture high quality data on efficacy and safety together with other information on standards of care and impact on Health Technology Assessment, such as number of hospitalizations or use of orthoses, devices etc.

The transition from a comprehensive academic dataset to a minimal, high-value mandatory dataset represents a methodological choice aimed at balancing data depth with scalability and inclusiveness. While the original registry included an extensive set of variables suitable for highly specialized centres, such an approach is difficult to sustain in a nationwide setting involving centres with heterogeneous resources and expertise.

The adoption of a core mandatory dataset ensures consistent data collection across all participating sites, reducing missingness and improving overall data completeness and comparability. At the same time, the availability of optional variables allows more detailed data capture in centres with greater capacity, preserving the richness of the dataset where feasible.

Importantly, this approach supports representativeness by enabling broader participation across centres and minimizing systematic exclusion of patients due to data collection burden. This trade-off between depth and coverage is consistent with regulatory perspectives emphasizing the importance of fit-for-purpose data sources that reflect real-world clinical practice while maintaining acceptable data quality standards.

Less restrictions were used when selecting the fields assessing safety with the implementation of mandatory use of MedDRA to provide detailed information on adverse events in a format that is compliant with regulators’ requests.

Other fields, including functional measures, became optional but all centres received the same training for the whole dataset. It is of interest that even if optional, details of functional measures were available in over 60% of patients and the number of centres providing these details is rapidly increasing.

All the centres in Italy agreed to be part of such a nationwide collaborative frame that was made possible thanks to funding from the companies. Collaboration with industry is structural in this domain, as although the registry does not require funding for the performance of the tests that are routinely performed in clinical practice, there are a number of other aspects from data entry, internal and external monitoring and maintenance of the platform that, because of their high standard, cannot be sustained by the centres without external support. This raises the issue not only of sustainability but also of the need for a structured Governance System to guarantee transparency in data usage. In order to do this, the Governance system that was part of the international collaboration was further reinforced increasing the number of advocacy groups, legal and ethicists representatives, in line with the regulators’ recommendations.

The process from ISMAR to ITASMAR was quite lengthy as the number of centres involved was much larger and Ethic Committee approval occurred within a time frame of over 18 months, with some centres having a delay in obtaining approval. Even with these limitations, we were able to have at least 12 month follow up data even in the centres with the delayed approval and to perform an analysis of data completeness not only for the baseline visit, which comprehends all the relevant background information, including genetic mutations and family and past history, but also for some of the follow up visits foreseen by the study protocol. The analysis shows that data completeness varied across all participating centres. Mandatory fields demonstrated consistently high completion rates ranging from 70 to 99%, whereas non-mandatory fields showed greater variability, with completion rates spanning from 34 to 100%.

A key consideration for the use of registry data in regulatory and policy decision-making is representativeness and generalisability. The ITASMAR registry was specifically designed to enhance representativeness by including all identified prescribing centres across Italy, encompassing both highly specialized academic sites and centres with varying levels of expertise and resources. This nationwide approach allows the registry to better reflect routine clinical practice and the heterogeneity of patient management compared to earlier academic-only datasets.

Furthermore, the inclusion criteria are deliberately broad, capturing all individuals with genetically confirmed 5q SMA irrespective of age, disease subtype, or treatment status, with a very low refusal rate (<1%). These features support the external validity of the data and its potential applicability to real-world populations encountered in clinical and regulatory settings.

However, some limitations should be acknowledged. Although nationwide, the registry is not strictly population-based and may not fully capture patients who are not followed in the involved centres. In addition, variability in data completeness for non-mandatory variables and differences in clinical practice across centres may introduce heterogeneity. Ongoing efforts to improve data harmonization, linkage with other data sources, and continued expansion of participation will further strengthen representativeness over time.

Regular online meetings and at least one in person meeting/year with retraining helped to motivate the centres and to ascertain that all the people involved were regularly retrained for performing examination, data entry and data management. Internal and external monitoring with routine feedback also proved to be essential to reduce missingness and improve timeliness.

Our experience suggests that it is feasible to expand highly specialized registries across multiple centres and keep pace with the need to provide accurate real word data on a large scale to better monitor the progression of SMA after the advent of the new therapies. A shared language and minimal core datasets, continuous training and transparent governance, and a more explicit interface with industry and regulators are essential prerequisites for meaningful, scalable, and reproducible learning from care. In SMA the expansion to multiple centers is also facilitated by the fact that the total number of centers is relatively small and it is also possible to monitor the adherence to the available care recommendations (38, 39).

The next phase will be defined by federation, methodical analytics, and progressive alignment with recognized qualification pathways where appropriate (34). The objective is to expand registries to strengthen their scientific and clinical value so that data captured in everyday practice can reliably inform decisions both at the bedside and the regulatory policy table.

From a regulatory perspective, the evolution of ITASMAR reflects key attributes required for real-world data sources to be considered fit for purpose. These include structured and standardized data collection, implementation of internationally recognized coding systems (e.g., MedDRA, medicinal product coding), robust governance frameworks, auditability through comprehensive audit trails, and multi-layered data quality assurance processes.

In this context, ITASMAR has the potential to support multiple regulatory and policy-relevant applications, including post-marketing safety surveillance, long-term effectiveness assessment, and the generation of external control data in settings where randomized clinical trials are not feasible or sufficient. Furthermore, the nationwide scope and inclusion of heterogeneous clinical settings enhance its value for health technology assessment and reimbursement decision-making.

Continued efforts toward methodological refinement, data harmonization, and alignment with regulatory qualification pathways will be essential to fully realize the potential of the registry as a source of regulatory-grade real-world evidence and secure funding for its maintenance.

## Data Availability

The original contributions presented in the study are included in the article/Supplementary material, further inquiries can be directed to the corresponding author.

## Group member of ITASMAc working group

The ITASMAc working group: Emilio Albamonte, Michela Catteruccia, Michele Tosi, Noemi Brolatti, Federica Trucco, Chiara Panicucci, Simone Morando, Ilaria cavallina, Giulio Gadaleta, Enrica Rolle, Giuliana Capece, Claudia Dosi, Gloria Pomè, Alice Gardani, Selvia Khalil, Francesca Benedetti, Elena Briganti, Gaia Scarpini, Esther Picillo, Maria Rosaria Manna, Ilaria Bitetti, Barbara Risi, Chiara Colombi, Giulia Gilberti, Nesaiba Allali, Rocco Liguori, Chiara Ticci, Elena Procopio, Martina De Maria, Dario Zoppi, Maria Elena Pessa, Paolo Alonge, Antonio Lauletta, Laura Fionda, Bianca Buchignani, Silvia Frosini, Gabriele Siciliano, Sara Lopriano, Francesca Torri, Stefano Scarparo, Maria Rosaria Vizzino, Maria Celeste Pirozzoli, Silvia Bonanno, Delia Gagliardi, Stefano Previtali, Antonella Longo, Eleonora Giacopuzzi Grigoli, Marta Cheli, Sophia Paolucci, Lavinia Fanelli, Sara Carnicella, Nicola Forcina, Roberto De Sanctis, Giulia Norcia, Giulia Stanca, Claudia Faini, Luca Teodorani, Stefano Parravicini, Maria Sframeli, Elena Sogus, Tommaso Fornasier.

## References

[1] Mercuri E Sumner CJ Muntoni F Darras BT Finkel RS. Spinal muscular atrophy. Nat Rev Dis Primers. (2022) 8:52. doi: 10.1038/s41572-022-00380-8 35927425

[2] Finkel RS Mercuri E Darras BT Connolly AM Kuntz NL Kirschner J . Nusinersen versus sham control in infantile-onset spinal muscular atrophy. N Engl J Med. (2017) 377:1723–32. doi: 10.1056/NEJMoa1702752, 29091570

[3] Mercuri E Darras BT Chiriboga CA Day JW Campbell C Connolly AM . Nusinersen versus sham control in later-onset spinal muscular atrophy. N Engl J Med. (2018) 378:625–35. doi: 10.1056/NEJMoa1710504, 29443664

[4] Mendell JR Al-Zaidy S Shell R Arnold WD Rodino-Klapac LR Prior TW . Single-dose gene-replacement therapy for spinal muscular atrophy. N Engl J Med. (2017) 377:1713–22. doi: 10.1056/NEJMoa1706198, 29091557

[5] Baranello G Darras BT Day JW Deconinck N Klein A Masson R . Risdiplam in type 1 spinal muscular atrophy. N Engl J Med. (2021) 384:915–23. doi: 10.1056/NEJMoa2009965, 33626251

[6] Mercuri E Baranello G Boespflug-Tanguy O De Waele L Goemans N Kirschner J . Risdiplam in types 2 and 3 spinal muscular atrophy: a randomised, placebo-controlled, dose-finding trial followed by 24 months of treatment. Eur J Neurol. (2022) 30:1945–56. doi: 10.1111/ene.15499, 35837793

[7] Boardman FK Sadler C Young PJ. Newborn genetic screening for spinal muscular atrophy in the Uk: the views of the general population. Mol Genet Genomic Med. (2018) 6:99–108. doi: 10.1002/mgg3.353, 29169204 PMC5823674

[8] Vill K Schwartz O Blaschek A Glaser D Nennstiel U Wirth B . Newborn screening for spinal muscular atrophy in Germany: clinical results after 2 years. Orphanet J Rare Dis. (2021) 16:153. doi: 10.1186/s13023-021-01783-8, 33789695 PMC8011100

[9] Balaji L Farrar MA Yiu EM Kariyawasam D. A state-of-the-art review of registries in spinal muscular atrophy: a valuable resource for clinical research. J Neuromuscul Dis. (2025) 12:312–29. doi: 10.1177/22143602241313113, 40033694 PMC13142879

[10] Puig-Ram C Segovia S Garcia-Uzquiano R Nungo Garzon NC Aragon-Gawinska K Garcia Romero M . Real-world data on spinal muscular atrophy in Spain: insights from over 500 individuals in the Cuidame project. J Neuromuscul Dis. (2025) 12:837–48. doi: 10.1177/22143602251361190, 40726133 PMC13142834

[11] Lusakowska A Jedrzejowska M Kaminska A Janiszewska K Grochowski P Zimowski J . Observation of the natural course of type 3 spinal muscular atrophy: data from the polish registry of spinal muscular atrophy. Orphanet J Rare Dis. (2021) 16:150. doi: 10.1186/s13023-021-01771-y, 33761963 PMC7992780

[12] Abbott L Main M Wolfe A Rohwer A Baranello G Munot P . Spinal presentations in children with type 1 spinal muscular atrophy on Nusinersen treatment across the Sma-Reach Uk network: a retrospective National Observational Study. BMJ Open. (2025) 15:e082240. doi: 10.1136/bmjopen-2023-082240, 39842910 PMC11784377

[13] Mercuri E Finkel R Scoto M Hall S Eaton S Rashid A . Development of an academic disease registry for spinal muscular atrophy. Neuromuscul Disord. (2019) 29:794–9. doi: 10.1016/j.nmd.2019.08.014, 31558335

[14] Pechmann A Konig K Bernert G Schachtrup K Schara U Schorling D . Smartcare - a platform to collect real-life outcome data of patients with spinal muscular atrophy. Orphanet J Rare Dis. (2019) 14:18. doi: 10.1186/s13023-019-0998-4, 30665421 PMC6341722

[15] Albamonte E Lizio A Coratti G Maggi L Pegoraro E Pane M . Patients on treatment with Risdiplam in Italy: challenges in the interpretation of the real-world data. Neurol Sci. (2025) 46:3839–49. doi: 10.1007/s10072-025-08125-7, 40153113

[16] Coratti G Bovis F Pera MC Scoto M Montes J Pasternak A . Determining minimal clinically important differences in the Hammersmith functional motor scale expanded for untreated spinal muscular atrophy patients: an international study. Eur J Neurol. (2024) 31:e16309. doi: 10.1111/ene.16309, 38656662 PMC11236020

[17] Coratti G Brogna C Norcia G Ricotti V Abbott L D'Amico A . Longitudinal natural history in Young boys with Duchenne muscular dystrophy. Neuromuscul Disord. (2019) 29:857–62. doi: 10.1016/j.nmd.2019.09.010, 31629611

[18] Coratti G Carmela Pera M Montes J Scoto M Pasternak A Bovis F . Revised upper limb module in type ii and iii spinal muscular atrophy: 24-month changes. Neuromuscul Disord. (2022) 32:36–42. doi: 10.1016/j.nmd.2021.10.009, 34980538

[19] Coratti G Lucibello S Pera MC Duong T Muni Lofra R Civitello M . Gain and loss of abilities in type ii Sma: a 12-month natural history study. Neuromuscul Disord. (2020) 30:765–71. doi: 10.1016/j.nmd.2020.07.004, 32893082

[20] Coratti G Messina S Lucibello S Pera MC Montes J Pasternak A . Clinical variability in spinal muscular atrophy type iii. Ann Neurol. (2020) 88:1109–17. doi: 10.1002/ana.25900, 32926458

[21] Coratti G Pane M Lucibello S Pera MC Pasternak A Montes J . Age related treatment effect in type ii spinal muscular atrophy pediatric patients treated with Nusinersen. Neuromuscul Disord. (2021) 31:596–602. doi: 10.1016/j.nmd.2021.03.012, 34099377

[22] Coratti G Pera MC Lucibello S Montes J Pasternak A Mayhew A . Age and baseline values predict 12 and 24-month functional changes in type 2 Sma. Neuromuscul Disord. (2020) 30:756–64. doi: 10.1016/j.nmd.2020.07.005, 32900576

[23] Coratti G Pera MC Montes J Pasternak A Scoto M Baranello G . Different trajectories in upper limb and gross motor function in spinal muscular atrophy. Muscle Nerve. (2021) 64:552–9. doi: 10.1002/mus.27384, 34327716 PMC9291175

[24] Pera MC Coratti G Mazzone ES Montes J Scoto M De Sanctis R . Revised upper limb module for spinal muscular atrophy: 12 month changes. Muscle Nerve. (2019) 59:426–30. doi: 10.1002/mus.26419, 30677148

[25] Sansone VA Coratti G Pera MC Pane M Messina S Salmin F . Sometimes they come Back: new and old spinal muscular atrophy adults in the era of Nusinersen. Eur J Neurol. (2021) 28:602–8. doi: 10.1111/ene.14567, 33012052

[26] Coratti G Ricci M Capasso A D'Amico A Sansone V Bruno C . Prevalence of spinal muscular atrophy in the era of disease-modifying therapies: an Italian Nationwide survey. Neurology. (2022) 100:522–8. doi: 10.1212/WNL.0000000000201654, 36460469 PMC10074458

[27] European MA. Guideline on Registry-Based Studies. Amsterdam: European Medicines Agency (2021).

[28] European MA. Reflection Paper on Use of Real-World Data in Noninterventional Studies to Generate Real-World Evidence for Regulatory Purposes. Amsterdam: European Medicines Agency (2025).

[29] European MA. Data Quality Framework for Eu Medicines Regulation: Application to Real-World Data. Amsterdam: European Medicines Agency (2024).

[30] European MA. Real-World Evidence Framework to Support Eu Regulatory Decision-Making: Report on the Experience Gained with Regulator-Led Studies from September 2021 to February 2023. Amsterdam: European Medicines Agency (2023).

[31] Brown EG Wood L Wood S. The medical dictionary for regulatory activities (Meddra). Drug Saf. (1999) 20:109–17. doi: 10.2165/00002018-199920020-00002, 10082069

[32] European Medicines A. (2026). Public Data from the Article 57 Database.

[33] Mercuri E Coratti G Cutrona C De Sanctis R Stanca G Cicala G . Development of the "Sma Nne," a short neonatal neurological examination for newborns with spinal muscular atrophy. Eur J Pediatr. (2025) 184:562. doi: 10.1007/s00431-025-06382-4, 40825903 PMC12360983

[34] Plueschke K Jonker C Kankanen H Vetter T Sepodes B Naehrlich L . Optimizing patient registries for regulatory decision making - key learnings from an Hma/Ema multistakeholder workshop. Clin Pharmacol Ther. (2025) 118:551–60. doi: 10.1002/cpt.3733, 40457718 PMC12355018

[35] Zebachi S Tanniou J Bakker E de Vries ST Di Bidino R Xoxi E . Navigating the real world: a scoping review of structured frameworks to effectively identify, evaluate, and select real-world data sources for fit-for-purpose studies. Clin Pharmacol Ther. (2025) 118:894–905. doi: 10.1002/cpt.3746, 40601391 PMC12439006

[36] FDA. Framework for Fda’s Real-World Evidence Program. Silver Spring, MD: FDA (2018).

[37] Bladen CL Thompson R Jackson JM Garland C Wegel C Ambrosini A . Mapping the differences in care for 5,000 spinal muscular atrophy patients, a survey of 24 National Registries in North America Australasia and Europe. J Neurol. (2014) 261:152–63. doi: 10.1007/s00415-013-7154-1, 24162038

[38] Mercuri E Finkel RS Muntoni F Wirth B Montes J Main M . Diagnosis and Management of Spinal Muscular Atrophy: part 1: recommendations for diagnosis, rehabilitation orthopedic and nutritional care. Neuromuscul Disord. (2018) 28:103–15. doi: 10.1016/j.nmd.2017.11.005, 29290580

[39] Finkel RS Mercuri E Meyer OH Simonds AK Schroth MK Graham RJ . Diagnosis and Management of Spinal Muscular Atrophy: part 2: pulmonary and acute care; medications, supplements and immunizations; other organ systems; and ethics. Neuromuscul Disord. (2018) 28:197–207. doi: 10.1016/j.nmd.2017.11.004, 29305137

