# Supplementary material for: Modifying disease registries to address the evolving field in rare diseases: the iSMAc/ITASMAc experience in spinal muscular atrophy
Source: Front Neurol. 2026 Jun 17;17:1833889. doi: 10.3389/fneur.2026.1833889 (PMC13318750; doi:10.3389/fneur.2026.1833889)
Supplement: Supplementary file 1 [file Data_Sheet_1.PDF]

Visit Type	N Vars	Complete	Average % Missing	Unknown	Median	Range Min	Max
**SMA Info**							
Baseline	9	96.0	2.3	1.8	98.9	87.8	100.0
Follow-Up	3	99.7	0.3	0.0	100.0	99.1	100.0
**Molecular Genetic Diagnosis**							
Baseline	9	96.0	2.3	1.8	98.9	87.8	100.0
**Growth**							
Baseline	3	76.7	14.3	8.9	72.6	72.4	85.2
Follow-Up	3	85.1	6.3	8.5	83.1	83.1	89.2
**Motor Function**							
Baseline	5	87.6	7.7	4.7	86.9	86.1	91.6
**Motor Function subfields**							
Baseline	23	93.1	6.9	0.0	100.0	23.8	100.0
**Nutrition**							
Baseline	3	98.6	0.1	1.2	98.9	97.0	100.0
Follow-Up	2	98.8	0.4	0.7	98.8	97.7	100.0
**Nutrition subfields**							
Baseline	14	99.2	0.0	0.8	100.0	90.7	100.0
Follow-Up	2	94.0	0.0	6.0	94.0	87.9	100.0
**DMTs**							
Baseline	5	99.2	0.2	0.6	99.8	98.0	99.8
Follow-Up	5	99.7	0.1	0.2	99.8	99.2	99.9
**DMTs subfieldss**							
Baseline	7	94.9	1.1	4.0	98.8	81.4	99.3
Follow-Up	7	96.0	1.0	2.9	98.8	82.0	100.0
**S/Adverse Events**							
Baseline	5	95.9	1.6	2.5	96.7	92.9	98.8
Follow-Up	5	99.5	0.1	0.4	99.5	99.2	99.7
**Medical subfieldss**							
Baseline	43	84.5	11.9	3.6	88.9	9.0	100.0
Follow-Up	49	84.4	13.5	2.1	93.5	4.8	100.0
**Scoliosis**							
Baseline	5	95.2	0.2	4.5	96.8	92.8	96.9
Follow-Up	5	97.2	0.1	2.6	98.4	93.2	99.2
**scoliosis subfieldss**							
Baseline	3	70.6	0.0	29.4	84.1	27.7	100.0
Follow-Up	3	75.2	0.1	24.7	92.8	32.7	100.0
**Vitamin D**							
Baseline	1	94.2	0.2	5.6	94.2	94.2	94.2
Follow-Up	1	97.2	0.0	2.8	97.2	97.2	97.2
**Vitamin D subfieldss**							
Baseline	2	99.8	0.2	0.0	99.8	99.8	99.8
Follow-Up	2	99.9	0.1	0.0	99.9	99.9	99.9
**Ventilation Assistance**							
Baseline	2	91.9	0.2	7.9	91.9	86.4	97.4
Follow-Up	3	98.0	0.4	1.5	98.8	96.1	99.2
**Ventilation Assistance subfieldss**							
Baseline	7	93.8	0.1	6.1	97.5	67.2	99.8
Follow-Up	7	93.5	0.2	6.2	97.5	63.3	100.0
**Ventilation**							
Baseline	3	99.0	0.2	0.7	99.5	97.8	99.7
**Ventilation subfieldss**							
Baseline	5	96.1	0.0	3.9	97.0	87.6	100.0
**Ventilation Treatments**							
Baseline	6	99.1	0.2	0.6	99.3	98.5	99.5
Follow-Up	6	99.7	0.2	0.1	99.7	99.6	100.0
**Ventilation Treatments subfieldss**							
Baseline	25	90.3	1.4	8.3	100.0	47.1	100.0
Follow-Up	25	91.4	1.6	7.0	99.5	45.8	100.0
**Rehabilitation**							
Baseline	3	90.9	7.0	2.1	87.7	87.5	97.5
Follow-Up	3	98.6	0.2	1.1	98.5	98.1	99.3
**Devices**							

Baseline	2	98.5	0.2	1.1	98.5	98.1	98.9
Follow-Up	2	99.2	0.1	0.6	99.2	99.2	99.3
**Devices subfieldss**							
Baseline	23	98.5	0.1	1.4	100.0	70.3	100.0
Follow-Up	23	98.4	0.3	1.3	100.0	70.2	100.0

Supplementary table 1. Data Completeness Summary by Sections and Visit Type for mandatory fields

Visit Type	N Vars	Average %			Range		
		Complete	Missing	Unknown	Median	Min	Max
**SMA Info**							
Baseline	1	94.2	5.8	0.0	94.2	94.2	94.2
**Molecular Genetic Diagnosis**							
Baseline	1	94.2	5.8	0.0	94.2	94.2	94.2
**Molecular Genetic Diagnosis Subfields**							
Baseline	1	94.2	5.8	0.0	94.2	94.2	94.2
**Gestational age**							
Baseline	1	94.2	5.8	0.0	94.2	94.2	94.2
**Growth**							
Baseline	2	34.2	36.5	29.3	34.2	32.1	36.4
Follow-Up	2	46.3	22.5	31.2	46.3	45.6	47.0
**Nutrition**							
Baseline	3	80.4	17.0	2.6	79.0	78.8	83.5
Follow-Up	2	82.9	16.0	1.1	82.9	82.9	82.9
**Nutrition subfields**							
Baseline	7	82.1	15.9	2.1	93.8	0.0	100.0
Follow-Up	6	91.3	4.2	4.5	94.5	69.6	100.0
**DMTs**							
Baseline	4	82.6	16.1	1.3	82.7	82.1	83.1
Follow-Up	4	87.0	12.6	0.4	87.0	86.8	87.2
**DMTs subfieldss**							
Baseline	12	67.7	15.5	16.8	65.2	50.0	90.0
Follow-Up	12	80.6	10.7	8.7	81.9	52.7	97.7
**S/Adverse Events**							
Baseline	4	71.2	27.7	1.1	77.2	52.5	77.9
Follow-Up	1	87.6	12.1	0.3	87.6	87.6	87.6
**Medical subfieldss**							
Baseline	10	96.0	0.6	3.4	99.1	83.1	100.0
Follow-Up	7	100.0	0.0	0.0	100.0	100.0	100.0
**Vitamin D**							
Baseline	3	53.8	43.9	2.3	78.9	0.5	81.9
Follow-Up	3	61.7	37.4	0.9	91.3	1.4	92.4
**Vitamin D subfieldss**							
Baseline	5	85.8	14.0	0.2	87.5	67.3	93.5
Follow-Up	5	92.8	7.0	0.1	97.6	73.7	97.7
**Ventilation Assistance**							
Baseline	1	75.2	16.1	8.7	75.2	75.2	75.2
Follow-Up	4	83.6	13.9	2.5	83.7	83.4	83.8
**Ventilation Assistance subfields**							
Baseline	3	74.4	25.6	0.0	83.3	55.5	84.5
Follow-Up	6	93.5	6.4	0.2	95.6	78.2	100.0
**Ventilation Treatments**							
Baseline	8	67.9	22.2	9.9	69.6	54.5	77.1
Follow-Up	5	74.7	17.0	8.3	75.9	64.3	82.9
**Ventilation Treatments Subfieldss**							
Baseline	8	67.9	22.2	9.9	69.6	54.5	77.1
Follow-Up	5	74.7	17.0	8.3	75.9	64.3	82.9
**Rehabilitation**							
Baseline	2	53.9	38.2	8.0	53.9	52.2	55.5
Follow-Up	2	73.9	22.3	3.9	73.9	73.2	74.6
**Rehabilitation subfields**							
Baseline	14	81.1	6.6	12.4	78.6	60.0	100.0
Follow-Up	14	86.4	3.2	10.3	90.0	59.3	99.2
**Devices**							
Baseline	5	54.0	45.2	0.8	49.9	34.3	99.0
Follow-Up	5	99.8	0.0	0.2	99.8	99.5	100.0
**Devices subfieldss**							
Baseline	2	100.0	0.0	0.0	100.0	100.0	100.0
Follow-Up	2	100.0	0.0	0.0	100.0	100.0	100.0
**Lab Measures**							
Baseline	2	72.8	21.7	5.5	72.8	72.0	73.6
Follow-Up	2	95.7	0.0	4.3	95.7	95.6	95.8

**Lab Measures subfields**							
Baseline	10	83.5	16.4	0.1	95.2	56.0	99.4
Follow-Up	10	85.4	14.5	0.0	99.0	45.1	99.9
**Outcome measures**							
Baseline	9	68.5	31.5	0.0	63.9	63.0	78.6
Follow-Up	9	81.3	18.7	0.0	78.6	77.5	89.7

Complete = non-NA and non-unknown values; Missing = NA values; Unknown = explicitly coded as unknown.

Supplementary table 2. Data Completeness Summary by Sections and Visit Type for non-mandatory fields
